# Epilepsy and Myasthenia Gravis: A Case Series

**DOI:** 10.3390/brainsci14090870

**Published:** 2024-08-28

**Authors:** Iñigo Oyarzun, Guillermo Hernández, Jacint Sala-Padró, Francisco Morandeira, Carlos Casasnovas, Mercè Falip

**Affiliations:** 1 Neurology Service, Hospital de Basurto, 48013 Bilbao, Spain; oyarzun.irazu@gmail.com; 2 Epilepsy Unit, Neurology Service, Bellvitge University Hospital, Neurological Diseases and Neurogenetics Group, Neuroscience Area, Institud d´Investigació Hospital de Bellvitge (IDIBELL), Hospitalet de Llobregat, 08908 Barcelona, Spain; guillermohpe@gmail.com (G.H.); jacint.sala@gmail.com (J.S.-P.); 3 Immunology Unit, Central Laboratory Department, Bellvitge University Hospital, Hospitalet de Llobregat, 08907 Barcelona, Spain; fmrego@bellvitgehospital.cat; 4 Neuromuscular Unit, Neurology Service, Bellvitge University Hospital, Neurological Diseases and Neurogenetics Group, Neuroscience Area, Institud d´Investigació Hospital de Bellvitge (IDIBELL), Hospitalet de Llobregat, 08908 Barcelona, Spain; carloscasasnovas@bellvitgehospital.cat

**Keywords:** myasthenia gravis, epilepsy, autoimmunity, comorbidity, focal epilepsy, selenium

## Abstract

The association between epilepsy and myasthenia gravis has rarely been reported, and when it has been reported, it has only been in a small case series. The aim of the present study was to report the frequency of epilepsy and myasthenia gravis and to describe a case series of patients with myasthenia gravis and epilepsy, focusing on their clinical characteristics and searching for a possible physiopathological mechanism. A retrospective, observational, adult center study was conducted in 2022. Patients were recruited from the database of the outpatient clinic of the Myasthenia Gravis and Epilepsy Unit of the Neurology Service, Hospital Universitari de Bellvitge. Five patients were included. The frequency of epilepsy in the myasthenia gravis cohort was 5/469 (1.1%), and the frequency of myasthenia gravis in the epilepsy cohort was 5/1432 (0.35%). All patients suffered from focal epilepsy, mainly temporo-central, which was drug-resistant in 3/5 Myasthenia gravis, which was generalized and with exacerbations in 3/5. Three patients were thymectomized (anatomopathology: thymic hyperplasia). Other autoimmune diseases were found in two (40%). Epilepsy onset preceded myasthenia gravis onset in all patients. Both diseases were considered autoimmune-related in 3/5, related to genetic predisposition due to altered innate immune system in 1/5, and due to chance or to treatment in 1/5. Epilepsy and myasthenia gravis are only infrequently associated. In adult patients, epilepsy onset precedes myasthenia onset in most cases. In some cases, epilepsy has an autoimmune etiology and coexists with other autoimmune conditions.

## 1. Introduction 

Epilepsy, or diseases that produce seizures, are common chronic brain disorders, with a prevalence of between 0.5 and 1%, and this prevalence is growing [1]. The etiology of epilepsy can be structural, infectious, genetic, metabolic, or immune, or a combination of these. Unfortunately, for around 30% of patients, the etiology remains unknown [1]. 

Two large populational studies have shown the risk of epilepsy in children and adults with autoimmune diseases compared to individuals without autoimmune diseases to be significantly higher, with myasthenia gravis being the fourth most common comorbid autoimmune condition in an American cohort [2,3]. 

Myasthenia gravis (MG) is an immune-mediated disease characterized by fatigable muscle weakness of the ocular, facial, bulbar, respiratory, and limb muscles. Nearly 80–85% of MG cases are associated with acetylcholine receptor antibodies (AChR-Abs) that destroy synaptic transmission across the neuromuscular junction [4]. Moreover, other autoantibodies have been identified in patients with MG, including Abs against muscle-specific tyrosine kinase (MuSK) and lipoprotein receptor-related protein 4 (LRP4). The thymus is affected in most patients with AChR-positive MG; approximately 70% of patients have thymic hyperplasia, 10% thymoma, and the remainder either normal or atrophic thymus. The incidence rate of MG increases with age from 4.2 to 18.9 per million person–years [4], with a prevalence between 5 and 24 cases per 100,000 people. From 13 to 25% of patients with MG suffer from other autoimmune diseases [5,6], with the most frequent being systemic autoimmune diseases: thyroid diseases, systemic lupus erythematosus, and vitiligo. Neuromyelitis optica, inflammatory myopathy, and autoimmune encephalitis are the most common neurological diseases [5]. 

The first reported case of coexistent MG and epilepsy data is from 1915, and the first published series is from 1958 [7]; since then, only a few studies have been published [8,9,10].

The aim of the present study was to report a case series of patients with MG and epilepsy and to describe their clinical characteristics in the search for a possible physiopathological mechanism. 

## 2. Material and Methods 

### 2.1. Patients

A retrospective, observational, single-adult-center study was conducted in 2022. Patients were recruited from the outpatient clinic of the MG and Epilepsy Units of the Hospital Universitari de Bellvitge (HUB). 

This study was approved by the ethical committee of HUB (PI10/00738). Written informed consent was obtained from all patients.

### 2.2. Definitions and Inclusion–Exclusion Criteria

#### 2.2.1. MG Definition [11]

The definition of MG includes documented myasthenia gravis symptoms plus one of these two criteria: (a) serum positivity for AChR ab or MuSK ab and (b) a pathological electrophysiological study, as well as single-fiber electromyography (SFEMG) or repetitive nerve stimulation (RepStim).

Pathological SFEMG: mean jitter >50 μs and/or more than 10% of pairs had jitter >55 μs. Pathological RepStim: a decrease of >10% between the first and fourth muscle action potential amplitudes (CMAPs).

#### 2.2.2. Epilepsy Definition [12]

The definition of epilepsy includes confirmed epilepsy based on clinical history and supportive electroencephalogram (EEG) and brain magnetic resonance imaging (MRI).

Supportive EEG included the presence of interictal epileptiform discharges (IEDs). Supporting MRI included highly epileptogenic lesions such as a cortical arteriovenous malformation. 

Seizures have been described according to the accepted ILAE criteria [13].

#### 2.2.3. Autoimmune-Associated Epilepsy [1,14]

Immune etiology is the direct result of an immune disorder in which seizures are a core symptom. Immune etiology can be contemplated when there is evidence of autoimmune-mediated central nervous system inflammation or the presence of neuronal autoantibodies that have already been related with epilepsy at high titers.

Patients with acute symptomatic seizures (ASSs) secondary to autoimmune encephalitis (AE) were excluded from the study [14]. To rule out the possibility of ASS, a minimum follow-up of 2 years from the epilepsy debut was required, as well as an MRI/FDG-PET excluding inflammation. 

### 2.3. Diagnostic Procedures 

#### 2.3.1. Neuroimaging Studies

Magnetic resonance imaging (MRI). In all patients, MRI scans were acquired using a Philips 1.5 or 3 Tesla MRI scanner (Intera, Philips Medical Systems, Amsterdam, The Netherlands) according to a standard protocol. 

Fluodeoxiglucose (FDG) PET studies.

In patients with pharmacoresistant epilepsy, FDG-PET scans were carried out. These were acquired on a Gemini TF 64 PET/CT scanner (Philips, Amsterdam, The Netherlands) according to a standard protocol.

#### 2.3.2. Immunological Analysis

An immunological battery test was carried out including onconeuronal antibodies (ab), ab against neuronal surface antigens (NSAs), and antibodies to glutamic acid decarboxylase (GAD ab), AchR ab, MusK ab, and LRP4 ab. Antibodies were determined in blood serum. Onconeuronal ab (Hu, Yo, Ma, Tr, amphyphysin, CV2/CRMP5, SOX-1, Zic-4) testing was performed in HUB using immunoblot. NSA ab included LGI1/contactin-2-associated protein (CASPR2), N-methyl-D-aspartate receptor (NMDAR), α-amino-3-hydroxy-5-methyl-4-isoxazolepropionic acid receptor (AMPAR), γ-aminobutyric acid receptor (GABA_B_R), and dipeptidyl-peptidase-like protein-6 (DPPX). NSA-abs were identified by indirect immunofluorescence in cells transfected with neuronal antigens. GAD65 ab was analyzed as described elsewhere [15]. 

#### 2.3.3. Electrophysiological Studies

A standard EEG with sleep deprivation was conducted in all patients on a 64-channel digital Deltamed (Natus Medical, Paris, France) or 32 XLTEK EEG brain monitor (Almevan-Natus Medical Europe, Planegg, Germany). In pharmacoresistant patients, prolonged video-EEG monitoring was carried out on a XLTEK EMU 128 (Almevan-Natus Medical Europe, Planegg, Germany).

An EMG with SFEMG and/or RepStim was performed in all patients on a Nicolet EDX^®^ EMG/NCS/EP/IOM (Almevan-Natus Medical Europe, Planegg, Germany). 

## 3. Results 

**Figure 1:**
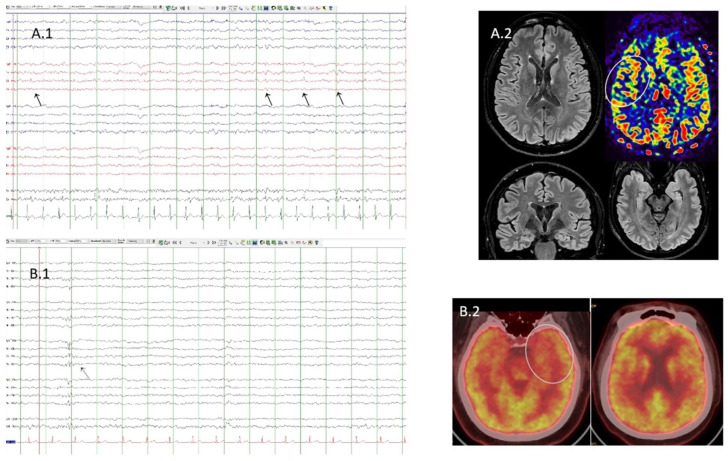
(**A.1**). Case 4. EEG with partial sleep deprivation. Bipolar montage. Presence of interictal epileptiform discharges (arrows) located in the right fronto-central area (**A.2**). Case 4. Flair sequences showing normal structural MRI. Arterial spin labeling sequence shows discrete hypoperfusion in the right operculo-insular area (white circle) (**B.1**). Case 1. Sleep EEG. Bipolar montage. Presence of interictal epileptiform discharge (black arrow) located in the left fronto-central area (**B.2**). Case 1. FDG-PET/TC showing left temporopolar hypometabolism (white circle).

Of 469 patients with MG included in the MG database, 5 (1.1%) patients also suffered from epilepsy. Of 1432 patients with epilepsy included in the epilepsy database, 5 (0.35%) also suffered from MG. The five identified are briefly described below (see also Table 1 and Figure 1).

### 3.1. Case 1

A fifty-year-old woman with drug-resistant epilepsy from the age of 17 presented with focal impaired awareness seizures (FIASs) sometimes evolving to bilateral tonic–clonic seizures. Her MRI was normal, and her EEG showed left interictal epileptiform discharges (IEDs). She is currently on levetiracetam (LEV) and lacosamide (LCM) without complete seizure control. In addition, she debuted with generalized MG at the age of 29, requiring a thymectomy; the anatomopathological result was thymic hyperplasia. She has also required immunosuppressive therapy and is currently on eculizumab and rituximab with difficult control of the disease. The two MG relapses and epilepsy worsening coincided. During the first two years with eculizumab, she suffered no MG relapses and no seizures. The immunological battery found positive AchR antibodies while the rest of the study, including GAD ab, was negative.

### 3.2. Case 2

A forty-seven-year-old woman presented with a nonlesional fronto-temporal lobe epilepsy onset at 25 years of age, and she was treated with carbamazepine (CBZ). At the age of 40 she reported seizure relapse, worsening of chronic headache, diplopia dyspnea, and muscle weakness and was diagnosed with PR3+ ANCA vasculitis with renal, pulmonary, and cerebral involvement, as well as the onset of generalized MG. Under treatment with mycophenolate mofetil and prednisone, symptoms were controlled. The immunological battery found positive AchR antibodies and low positive GAD ab (8 UI, normal values < 5UI), as well as positive PR3 ab (204–346 Karb·u/L).

### 3.3. Case 3

This case details a thirty-two-year-old man who suffered from a new onset of refractory status epilepticus (NORSE) at the age of 11. Immediately afterwards, he developed drug-resistant multifocal epilepsy. EEG recorded seizures starting independently from both frontocentral areas. Seizures started with a sensation of auditory plugging and anarthria. MRI and FDG-PET were normal. He has been on LEV, LCM, topiramate (TPM), clobazam (CBM), brivaracetam (BRV), and perampanel (PER) and is now on LCM-CBM and cenobamate, and he is still suffering from weekly seizures. He debuted with myasthenia gravis at the age of 25. He was thymectomized and treated with prednisone and azathioprine with complete control of symptoms. The immunological battery found positive AchR without neuronal antibodies including GAD ab.

### 3.4. Case 4

A sixty-nine-year-old woman presented with well-controlled right temporal lobe epilepsy onset at 41 with a nocturnal bilateral tonic–clonic seizure under treatment with valproic acid (VPA). Her MRI was normal. Seronegative ocular myasthenia gravis was diagnosed at 46 and was completely controlled without treatment. She has also suffered diabetes mellitus since age 52. Apart from GAD ab (85.4000 UI titres), the rest of the immunological battery was negative. 

### 3.5. Case 5

This case details a fifty-six-year-old woman with focal epilepsy due to right fronto-temporo-parietal arteriovenous malformation requiring as many as 28 embolizations. Drug-resistant epilepsy began at the age of 16. She had been treated with CBZ and phenobarbital (PB) for years with good control, and afterwards with LCM due to side effects (osteoporosis and hypercholesterolemia), which was changed to LEV. She suffered onset of generalized myasthenia gravis at the age of 50, just a few weeks after beginning treatment with LCM. She was thymectomized (thymic hyperplasia) and treated with prednisone and IVIG. The immunological battery found positive AchR ab. 

## 4. Discussion

In the present study, the frequency of epilepsy in the adult MG cohort was 1.1%, similar to the general population prevalence, but the frequency of MG in the epilepsy cohort was 0.4%, higher than the prevalence of MG in the general population [4] and similar to a populational study with more than 2 million people in which the prevalence of epilepsy in patients with MG was 0.4% [2]. Other series such as Di Stefano [6] and Badurska [8] have reported an even greater frequency (3.4 and 7%) of epilepsy in patients with MG. A possible reason for this difference is the population in the study; we included adult patients with epilepsy and excluded isolated seizures or ASS. Nonetheless, our study also suggests that people with epilepsy are at greater risk than the general population suffering from MG. 

Focusing on clinical characteristics of epilepsy in our series, all patients suffered focal epilepsy, which was nonlesional in four of them. Seizure semiology and EEG characteristics suggested a periopercular or temporo-insular origin in four patients, as reported previously [9]. Furthermore, MG was generalized with exacerbations in four of the patients and related to a thymic hyperplasia in all the thymectomized patients. 

Considering the association of MG and epilepsy, epilepsy onset preceded MG onset in all patients. Other authors have found the same timeline association [8,10,16]. In contrast to this, some patients with thymoma who developed a paraneoplastic AE suffered with myasthenia and ASS, with seizure onset preceding the myasthenic symptoms [5]. None of the other patients had thymoma or suffered from an AE.

Many hypotheses have been proposed to explain the association of MG and epilepsy [8,16]. The one that we consider the strongest is that these diseases are a reflection of the activation of a common autoimmune cascade, as in patients 2 and 4. Both patients suffer with autoimmune-associated epilepsy, patient 2 PR3+ ANCA vasculitis with cerebral involvement, and patient 4 GAD ab associated epilepsy. 

Other hypotheses suggest that the MG and epilepsy association is a reflection of a previously damaged autoimmune system as in patient 3, who had suffered a NORSE when he was a child perhaps due to a genetic condition; MG has been reported in patients with familiar Mediterranean gene mutations associated with MORVAN´s syndrome. In this case report, EEG was abnormal with signs of encephalopathy but without seizures [17].

Another hypothesis that might explain the association in patient 5 is that antiseizure drugs (ASDs), specifically LCM, may alter the neuromuscular junction [8] or trigger an autoimmune response in a susceptible individual, inducing MG in the same way that they induce systemic lupus erythematosus-like syndromes [18]. The involved ASDs include the following: CBZ [18], PHT [19], and trimethadione [20]. Finally, in patient 5, the association of the two diseases may also have been produced by chance. 

Interestingly, the trace element selenium (Se) could be a link between autoimmune disease predisposition and having suffered a NORSE or being chronically treated with ASDs. This element is essential for normal functioning of the immune system. An active immune system with elevated cytokine levels exerts a suppressive effect on hepatic selenoprotein biosynthesis. A severe acute or chronic Se deficit disturbs the immune system, irrespective of the underlying reason for it (e.g., infection) [21]. Furthermore, epilepsy, ASDs, and immunomodulatory treatment may affect the homeostasis of several trace metal levels [22]. Specifically, VPA can promote the clearance of copper, selenium, and zinc, reducing the synthesis of free-radical-scavenging enzymes [23]. There is no information about other ASDs. 

In addition, immunomodulatory treatment (plasmapheresis) can reduce serum levels of some ASDs (clobazam, LEV, and TPM) producing seizure relapse. This is a possible explanation for the coexistence of MG and seizure relapses in patient 1 [16,24].

Regarding treatment, one of our patients (patient 1) was treated with eculizumab with complete MG symptoms and seizure control for two years. Eculizumab is an approved treatment for MG with positive AChR-Abs. Another recently approved treatment for seropositive MG is efgartigimod. Efgartigimod is a neonatal Fc receptor (FcRn) blocker designed to reduce pathogenic IgG autoantibody levels that have shown efficacy in MG and some GAD ab diseases such as stiff-person syndrome, also producing a decrease in serum GAD ab [25,26]. Unfortunately, GAD ab in GAD ab-associated epilepsy is likely not pathogenic, and neuronal damage is produced mainly by infiltrating cytotoxic T lymphocytes [27].

This study has several limitations: it is a retrospective and chart review study so selection bias and less-than-accurate recordkeeping may be present in the cases of a small number of reviewed patients. Also, we did not measure trace elements. 

## 5. Conclusions

Epilepsy and myasthenia gravis, although infrequently associated, are comorbid conditions. In adult patients, epilepsy onset precedes myasthenia onset in most cases. Focal epilepsy involves a widespread area, including the temporal lobe. In some cases, both diseases are autoimmune and coexist with other autoimmune conditions; in other cases, treatment (antiseizure drugs and immunomodulatory treatment) may induce a relapse of the other disease. 

## Figures and Tables

**Table 1:** Clinical characteristics of the included patients.

Patient nº	Sex (M/F)	Epilepsy Onset	MG Onset	Epilepsy Localization	Myasthenia Type	Epilepsy Etiology	MRI/FDG-PET	Epilepsy Semiology	ASDs	Myasthenia Treatment	Thymecmotized	RepStim	SFEMG	Antibodies	Related Conditions
1	F	17	29	Fronto-Temporal	Generalized IVb	Unknown	Nonlesional L. mesiotemporal h.	Warm sensation-FIAS	LEV, LCM	Eculizumab + Rituximab	Yes	Pathological	Pathological	AchR	
2	F	25	40	Right Fronto-central (Seizure recorded)	Generalized IIIa	Autoimmune	Nonlesional R. mesiotemporal h.	Hypersalivation-FIAS	CBZ	Mycophenolate mofetil + prednisone + IVIG	No	Pathological	Pathological	AchR, PR3, GAD low titers	PR3+ ANCA vasculitis
3	M	11	25	Bilateral fronto-central (Seizures recorded)	Generalized IIa	Post NORSE	Nonlesional	Anarthria+ sensation of auditory plugging FIAS	LCM, CLB BRV, CNB	Prednisone + azatioprine	Yes	Pathological	Pathological	AchR	N.O.R.S.E as debut
4	F	41	46	Left fronto-temporal	Ocular I	Autoimmune	Nonlesional	Nocturnal BTCS	VPA	No	No	Nonpathological	Not performed	GAD high titers	DM-LADA, Hypothyroidism
5	F	16	50	Focal (fronto-temporal) epilepsy	Generalized IIIa	Vascular	Fronto-temporo-parietal arteriovenous malformation	FAS (right arm clonias)	LEV LCM worsened myasthenia	Prednisone IVIG for relapses	Yes	Pathological	Not performed	AchR	Hypothyroidism

FIAS: focal impaired awareness seizure, FAS: focal awareness seizure (motor), M: male, MRI: magnetic resonance image, F: female, NORSE: new onset refractory status epilepticus, IVIG: immunoglobulins, CBZ: carbamazepine, LCM: lacosamide, LEV: levetiracetam, VPA: valproate, BRV: brivaracetam, R: right, L: left, h: hypometabolism, BTCS: bilateral tonic–clonic seizure, DM-LADA: late-adult-onset diabetes mellitus, REpStim: repetitive stimulation in electromyography (EMG), SFEMG: single-fiber EMG.

## Data Availability

Data that support the findings of this study are available from the corresponding author upon reasonable request.
